# Additional Vitamin and Mineral Support for Patients with Severe Burns: A Nationwide Experience from a Catastrophic Color-Dust Explosion Event in Taiwan

**DOI:** 10.3390/nu10111782

**Published:** 2018-11-16

**Authors:** Li-Ru Chen, Bing-Shiang Yang, Chih-Ning Chang, Chia-Meng Yu, Kuo-Hu Chen

**Affiliations:** 1 Department of Physical Medicine and Rehabilitation, Mackay Memorial Hospital, Taipei 10449, Taiwan; gracealex168@gmail.com (L.-R.C.); genine0607@gamil.com (C.-N.C.); 2 Department of Mechanical Engineering, National Chiao-Tung University, Hsinchu 300, Taiwan; bsyang@mail.nctu.edu.tw; 3 Division of Plastic and Reconstructive Surgery, Department of Surgery, MacKay Memorial Hospital, Taipei 10449, Taiwan; defense@mmh.org.tw; 4 Department of Obstetrics and Gynecology, Taipei Tzu-Chi Hospital, The Buddhist Tzu-Chi Medical Foundation, Taipei 970, Taiwan; 5 School of Medicine, Tzu-Chi University, Hualien 970, Taiwan

**Keywords:** vitamin, mineral, burns, nutritional support

## Abstract

Major burn injuries, which encompass ≥20% of the total body surface area (TBSA), are the most severe form of trauma because of the stress response they provoke, which includes hypermetabolism, muscle wasting, and stress-induced diabetes. In 2015, a color-dust explosion disaster occurred in the Formosa Fun Coast of Taiwan and injured 499 people, who were transferred via a nationwide emergency delivery system. Some recommendations are currently available regarding vitamin and mineral support for wound healing and recovery in severe burns, but there is a lack of evidence to confirm the benefits. Thus, the current study aimed to investigate the effects of additional vitamin and mineral support for patients with severe burn injuries. Sixty-one hospitalized individuals with major burns (full thickness and ≥20% TBSA) were classified into the supplement (*n* = 30) and control (*n* = 31) groups, according to whether they received supplementation with additional vitamins, calcium, and magnesium. There were significant differences between the supplement and control groups in the incidence of wound infection (30.0% vs. 77.4%, *p* < 0.001), sepsis (13.3% vs. 41.9%, *p* = 0.021), and hospitalization days (51.80 vs. 76.81, *p* = 0.025). After adjustment, logistic regression analysis revealed that, compared to those in the control group, patients in the supplement group had a lower risk for wound infection (OR 0.11; 95% CI 0.03–0.43; *p* = 0.002) and sepsis (OR 0.09; 95% CI 0.01–0.61; *p* = 0.014). Supplementation of multiple vitamins, calcium, and magnesium reduced the risk of wound infection and sepsis, shortened the time of hospitalization, and can be considered for use in major burns.

## 1. Introduction

A major burn injury, which encompasses more than 20% of the total body surface area (TBSA), is the most severe form of trauma or critical illness in terms of the aggravating stress response it provokes, which includes hypermetabolism, muscle wasting, and stress-induced diabetes [1,2,3,4,5,6,7,8]. It has been shown that in patients with burns affecting over 30% of their TBSA, metabolic rate is 40–80% above normal in the first few months after burn injury [1,2,8]. There is notable muscle wasting due to accelerated proteolysis, along with low serum vitamin and mineral status [2,3]. In such patients, other systemic disturbances include physiological perturbations, impaired immunity, and massive fluid shifts, all of which can significantly affect postburn recovery and outcomes [2,7].

**Figure 1:**
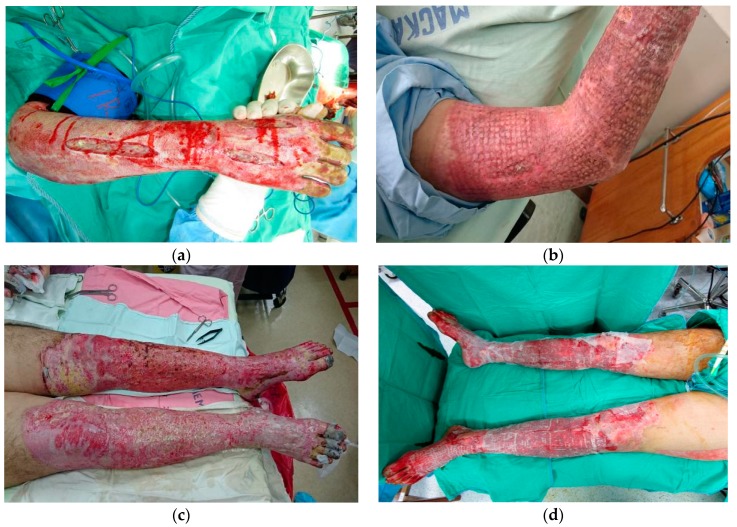
(**a**) Severe burn of the right hand, after escharotomy; (**b**) burn injury of the upper limb, status post-skin graft; (**c**) severe burn of the lower limbs with local necrosis; (**d**) burn injury of the lower limbs, after wound dressing.

In 2015, a color-dust explosion disaster in the Formosa Fun Coast of Taiwan injured 499 people. Most of the victims were young, and most experienced burns after the explosion [9]. During the Color Play Asia festival, a large deflagration occurred when a cloud of color-dust ignited after being discharged from the stage onto a crowd of 1000 people during a concert. Some of the crowd inhaled the color-dust powder, which caused severe respiratory problems. The color-dust, made of corn starch powder, caught fire along the ground, resulting in direct burns to the victims, mainly to their torsos and limbs (Figure 1). Many of the victims’ skin was sloughing off when touched. The victims were transferred to burn units in medical centers in Taiwan for acute burn care and further management. According to the offical statistical report of the Ministry of Health and Welfare, Taiwan, Republic of China, the average TBSA of the burns for all 499 domestic and foreign victims in this catastrophic event was 44%, and there were 248 victims with major burns of more than 40% TBSA [10]. Due to a nationwide delivery system of emergency and acute medical care, only 15 deaths were ultimately caused by the catastrophic color-dust explosion. Our research revealed that active rehabilitation therapy in survivors has been proven helpful for restoring normal function and for preventing limitation of activities and functional impairment [11]. Nevertheless, aggressive burn care with fluid and nutritional supplementation; appropriate use of analgesics and antibiotics; and surgeries, including wound debridement and skin grafts, is critical for patients in the early stages of major burn injury.

Current research has shown that enhanced nutritional support is essential for wound healing and physical recovery in patients with severe burns [4,5,6,8]. The aforementioned general support strategies, including high-calorie diet, fluid, and albumin [12] supplements, are commonly used to provide nutritional sources for such patients. Poor nutritional status or severe underfeeding of burned patients has been associated with muscle wasting, delayed wound healing, greater susceptibility to infection, and even death [4,5,8,13]. Based on the viewpoint that micronutrients (vitamins and minerals) play an essential role in antioxidant defense, and that a deficiency will amplify the metabolic perturbations and ongoing catabolism already induced by the burns, there is a rationale for supplementation with micronutrients [2,5]. Some recommendations are available for the supplementation of vitamins and trace elements after a burn injury [2,3,14]. However, there is a lack of evidence to confirm the benefits of vitamin and mineral support for wound repair, healing, and physical recovery in patients with severe burns. To date, important questions about macronutrient composition of enteral formulas, feeding modalities, personalized feeding regimens, and long-term outpatient nutritional support remain unanswered [1]. Currently, there are several areas in which consistency is not seen, such as the fat and carbohydrate content of enteral formulas, indications for the use of total parenteral nutrition, and the most appropriate strategies for perioperative feeding [3,4,8,13]. This reflects, to a great extent, inconclusive research outcomes. In particular, little progress has been made to further our understanding of the roles that advanced or additional micronutrients have in the recovery from burns [1,3]. Thus, this study aimed to investigate the effects of additional vitamin and mineral support for patients with severe burns during this nationwide disaster.

## 2. Materials and Methods

This longitudinal retrospective cohort study was conducted in a tertiary teaching hospital with a burn center. The study was approved by the Institutional Review Board of the study hospital (approval number: 16MMHIS005e), followed the principles set forth in the Declaration of Helsinki, and met the guidelines of the responsible governmental agency.

Individuals who sustained a major burn injury (full thickness and ≥20% TBSA) in this catastrophic event and consequently required hospitalization for acute and intensive care were included in the study. All eligible patients in the burn center were enrolled in the study during their admissions in order to decrease selection bias. Basic information, including age, body mass index (BMI), and sex were collected on admission. The depth (degree) and TBSA of the each patient’s burns were evaluated by the reconstructive and plastic surgeons. After admission, all patients received aggressive burn care, with respiratory intubation, fluid and general nutritional supplementation, albumin infusion according to serum albumin levels, analgesia, and antibiotics, as appropriate. After stabilization, all patients in the burn center underwent several wound debridements and skin grafts (e.g., split thickness skin grafts), depending on their physical condition and the TBSA of their burns. Wound repair, wound infection, and sepsis were assessed and managed by reconstructive and plastic surgeons belonging to the same team within the study hospital. Wound infection is defined as local infection involving skin, subcutaneous tissue, or deep tissue at the site of injury or surgical wound, with findings including purulent discharge, localized swelling, redness/heat, delayed wound healing, or positive wound culture results. Sepsis is the body’s overwhelming and life-threatening response to systemic infection, and can lead to tissue damage, organ failure, and death. It is clinically diagnosed with the findings of organ dysfunction, fever, tachycardia, tachypnea, and/or leukocytosis.

The supplement (intervention) and control groups were allocated based on which of the two burn units (within the burn center of the study hospital) the patients were sent to. Patients were delivered and admitted to burn units via a nationwide emergency delivery system, rather than being allocated or assigned according to the patients’ or surgeons’ preference. Patients in one burn unit were given additional vitamin and mineral support (the supplement group), and patients in the other burn unit were not (the control group), according to the different protocols of the two burn units.

During the first two weeks of hospitalization, daily vitamins were administered to those in the supplement group, including vitamin A 6600 IU, vitamin B1 (thiamine) 100 mg, vitamin B6 (pyridoxine) 200 mg, vitamin B12 2000 mcg, vitamin C (ascorbic acid) 100 mg, vitamin D 0.01 mg (400 IU), and vitamin E (dl-α-tocopheryl acetate) 20 mg. Mineral supplementation with calcium and magnesium was provided by the administration of calcium chloride 2%, 20 mL/amp, and magnesium sulfate injection 10%, 20 mL/amp, adjusted according to serum calcium and magnesium levels after biochemistry examinations. On the other hand, individuals in the control group did not receive additional vitamin and mineral support. Otherwise, all the patients in both groups received the same treatment (as outlined in the previous paragraph) from the same acute burn care team.

Under SPSS 19.0 (SPSS, Inc., Chicago, IL, USA), descriptive statistics, Chi-square (χ^2^) test, Fisher’s exact test, and Student *t*-test were performed to compare the characteristics and outcomes of the individuals in both groups. Logistic regression analysis was performed to assess the risk for various outcomes, including wound infection, sepsis, and hospitalization days, by reporting odds ratios (ORs) and 95% confidence intervals (CIs) after adjusting for age, BMI, sex, and the TBSA of the burns.

## 3. Results

**Figure 2:**
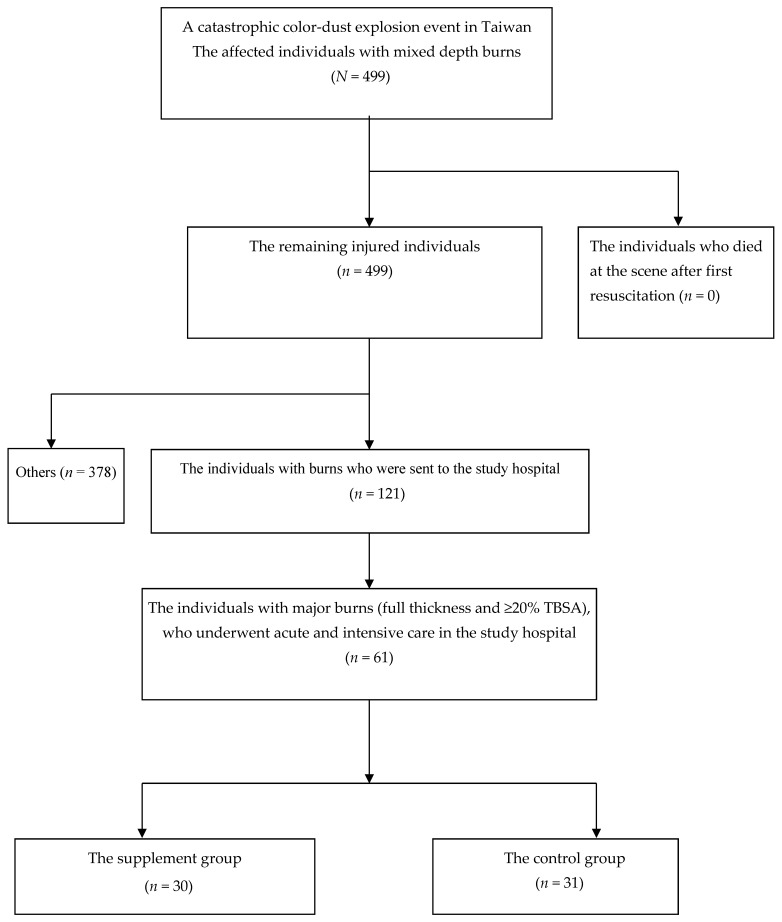
A flow diagram for patients with major burns in the catastrophic color-dust explosion event.

A flow diagram for patients with major burns in the catastrophic color-dust explosion event is shown in Figure 2. Through a nationwide medical system, the 499 patients were referred for further emergency treatment and acute medical care. Among them, 121 patients were sent to the study hospital, and 61 of these had major burns (full thickness and ≥20% TBSA), requiring hospitalization for acute and intensive care. Based on whether or not they received supplementation with additional vitamins and minerals, the individuals were classified into the supplement group (*n* = 30) and the control group (*n* = 31).

The characteristics and outcomes for patients in the supplement and control groups are presented and compared in Table 1. In both groups, most patients were young (21.57 vs. 23.45 years old for supplement and control groups, respectively), female (63.3% vs. 58.1%), and had a normal BMI (20.93 vs. 21.64). For patients with major burns, there were no significant differences in age, BMI, sex, or the average TBSA of burns (all *p* > 0.05) between the two groups. There were no deaths in the supplement group (0/30), and one death occurred in the control group (1/31). During a 3-year follow-up after discharge, no further patient deaths occurred in either group. Between the supplement and control groups, there were significant differences in the incidence of wound infections (30.0% vs. 77.4%, *p* < 0.001), sepsis (13.3% vs. 41.9%, *p* = 0.021), and hospitalization days (51.80 vs. 76.81, *p* = 0.025). Further statistical analyses comparing the risks of these outcomes are shown in Table 2. After adjustment, logistic regression analysis revealed that patients in the supplement group had a lower risk for wound infections compared with those in the control group (OR 0.11; 95% CI 0.03–0.43; *p* = 0.002). Patients in the supplement group also had a lower risk for sepsis compared with those in the control group (OR 0.09; 95% CI 0.01–0.61; *p* = 0.014), besides the additional risk factor represented by the TBSA of the burns (OR 1.09; 95% CI 1.03–1.16; *p* = 0.006).

## 4. Discussion

The elements that affect patient recovery from a major burn injury are multifactorial. However, nutritional support plays a critical role in the overall recovery of patients, which can be assessed by several indicators including wound healing, wound infection [13,14], sepsis, and number of days of hospitalization [4,7,14]. The results of the current study revealed that patients in the control group had a higher risk for wound infections (OR 9.46; 95% CI 2.31–38.69) and sepsis (OR 11.37; 95% CI 1.63–79.20). Without additional vitamin and mineral supplementation, patients had a nearly tenfold risk for wound infection and sepsis, as well as longer hospitalizations. In other words, supplementation of micronutrients, including multiple vitamins, calcium, and magnesium, reduced the risk of wound infection and sepsis, shortened the days of hospitalization, and can be considered for use in the acute care of patients with major burns.

Vitamin A is necessary for epithelial integrity and for optimal wound healing [5,8,15,16]. Many aspects of the immune response, including mucus production, phagocytosis, and humoral and cell-mediated immunity are depressed in patients with vitamin A deficiency [15,16]. Partly through the regulation of calcium and phosphorus metabolism, vitamin D has extra immunoregulatory functions [15,17]. Because long-term hospitalization and wound coverage with dressings may limit UV-light-mediated vitamin D formation, patients with major burns are at risk of vitamin D deficiency [15,18,19]. Vitamin E has many roles, including serving as an antioxidant, providing some protection from lung injury following thermal trauma, enhancing the immune response, and improving acute burn healing [15]. Vitamins B1, B6, and B12 are associated with cellular energy metabolism, amino acid metabolism and protein synthesis, and folic acid metabolism, respectively [15]. Vitamin C (ascorbic acid) is involved in the formation of collagen and the healing of skin, and functions as an antioxidant, or free-radical scavenger, to protect tissues from superoxide injury [3,5,8,15,20,21]. Minerals such as calcium and magnesium have important effects on nerves and muscles. A previous study of ours clarified that calcium and vitamin D deficiencies are associated with osteoporosis and fractures [22]. Hypocalcemia and subsequent osteoporosis are common in patients with major burns due to urinary calcium wasting and vitamin D insufficiency [8,23,24]. At present, recommendations regarding the timing and daily dosage of micronutrient supplements are inconsistent [2]. Some studies suggest supplements of two to three times the recommended dietary allowance (RDA) [2,3], while some other studies do not [5,6,7,19].

Our study provided evidence that additional micronutrients, including supplements of multiple vitamins, calcium, and magnesium, are beneficial for the recovery of patients suffering from burn injuries. The benefits of supplementing multiple vitamins, calcium, and magnesium in patients with major burns can possibly be attributed to the extensive loss of micronutrients associated with burn injuries and the increased need for tissue reconstruction during the repair period. Due to skin breakdown and tissue necrosis, there is a large amount of fluid and protein loss, along with micronutrient consumption and depletion [3,7,25]. The hypermetabolic state and subsequent tissue repair in patients who have sustained burn injuries calls for an increased input of micronutrients. Acting as coenzymes for individual metabolism, tissue repair, and activation of the immune system, vitamins may play a key role in wound healing, as well as in preventing wound infection and sepsis. Research has shown that decreased levels of vitamins A, C, and D adversely affect wound healing and skeletal, neuromuscular, and immune system function, and that oxidative stress contributes to secondary tissue damage and further impairs immune function [5]. Vitamin A improves wound healing time due to its effect on epithelial growth, whereas vitamin C facilitates the synthesis and cross-linking of collagen, protecting tissues from superoxide injury [5,6,8,15]. Vitamin D supplementation may also be helpful for the recovery of patients with burn injuries [17,18,19]: when combined with optimized calcium intake, it demonstrated positive effects on muscle health [24]. While there is no gold standard for the optimal dosage of vitamins in burn patients [5,6,17], we believe that a dosage higher than the daily RDA is necessary for effective supplementation and actions. This view is similar to one emerging from another report, which advocates providing at least the RDA [6]. More research is needed to determine the optimal dosage and timing for vitamin and mineral supplementation in patients with major burn injuries.

For eligible patients with major burns (full thickness and ≥20% TBSA) in the catastrophic event, acute medical care was provided by reconstructive and plastic surgeons belonging to the same team within the study hospital. Wound repair, wound infection, and sepsis were assessed and managed by the same reconstructive and plastic surgeons. Patients with severe burns were sent to the two burn units according to assignment by the nationwide emergency delivery system. Although the current study was not randomized, the patient characteristics, including age, BMI, sex, and the average TBSA of burns (Table 1) in both groups were compared to ensure the outcomes were mainly affected by the intervention (additional vitamin and mineral support) rather than by the patient characteristics. The comparisons in Table 1 showed there were no significant differences in age, BMI, sex, or the average TBSA of burns (all *p* > 0.05) between the two groups.

Our study had several strengths, including a large sample size, high level of patient homogeneity, and strict criteria for comparisons between groups. Owing to uncertainties regarding the occurrence and timing of burn injuries, sample sizes of burn patients over the period of time of a study are usually too small to make further inferences. The sample size of the current study was large compared to existing studies of its kind that investigate the role of additional nutritional support for patients with major burns. Furthermore, all patients in the study came from the same catastrophic color-dust explosion event, were young, and were sent to the same hospital (i.e., the study hospital). Except for differences in TBSA and the depth of burn injuries, due to the differing distance of each patient from the source of the deflagration, all patients sustained similar burn injuries and were transferred by the same nationwide emergency delivery system. Therefore, the homogeneity of the patients was high, due to their similar backgrounds. Moreover, all patients in both groups underwent the same treatment from the same medical team for burn care, except for the use of additional nutritional supplements in one group. All biases resulting from the samples, the selection, the burn care, and the caregivers were minimized by the methods and criteria used. Therefore, our results are robust due to the minimization of possible errors that originate from the research subjects and methods.

The current study has several inherent limitations, despite its strengths. First, the study was conducted in a tertiary teaching hospital, and the patients with burn injuries were retrieved from a catastrophic explosion event. Thus, generalization of the conclusions to other patients treated in smaller hospitals or local clinics, or to patients who sustain other types of burns (e.g., electric or chemical burn injuries) should be made with caution. Moreover, some demographic characteristics, such as race, previous smoking habits, social status, and economic status, were not accounted for in the current study. Thus, we were unable to investigate the contributing influences of these factors. Patients in the two different burn units received care from the same team of physicians, nutritionists, infection specialists, and social workers. However, each unit had different nursing staff, and potential differences in care resulting from this may be one limitation of the study. Except for different micronutrient supplements, the policies for medical care, including infection control practices, use of antimicrobials, frequency of dressing changes, use of skin substitutes, time and completeness of burn excision, and calorie/protein feeding regimens, were the same for both units. As far as we know, there were no outbreaks of nosocomial infection in either of the units. However, the different environments of the two burn units, interpersonal differences among medical staff who provided care, and differences between nursing staff may partially account for differences in wound infection and sepsis in both groups. These aforementioned factors were beyond our control, and may have acted as confounders that biased the results of the current study. Finally, in this longitudinal study, some factors that varied with time and changes in medical policies could not be controlled.

## 5. Conclusions

Without additional vitamin and mineral support, patients had a nearly tenfold risk for the occurrence of wound infection and sepsis, as well as longer hospitalization. The supplementation of multiple vitamins, calcium, and magnesium reduced the risk of wound infection and sepsis and shortened hospitalization time, and can be considered for use in the acute care of patients with major burns. Even though this study provides evidence and is an exciting step towards improving the outcomes of burn patients, much about additional nutritional support for burn patients remains unknown and warrants further investigation.

## Figures and Tables

**Table 1:** Comparison of patient characteristics and outcomes between the supplement group and the control group.

	Supplement Group (*n* = 30)		Control Group (*n* = 31)		Statistics
	*N*	%	*N*	%	*p*-Value
	(mean ± SD)		(mean ± SD)		
**Characteristics**					
**Age** **(years** **)**	21.57 ± 2.82		23.45 ± 4.67		0.062
**BMI (kg/m^2^)**	20.93 ± 1.77		21.64 ± 1.60		0.102
**Sex (gender)**					0.674
Male	11	36.7	13	41.9	
Female	19	63.3	18	58.1	
**Average TBSA of burns (%)**	53.37 ± 20.29		51.55 ± 20.80		0.731
20–40%	8	26.7	10	32.3	
40–60%	9	30.0	10	32.3	
>60%	13	43.3	11	35.5	
**Outcomes**					
**Death after hospitalization**					
No	30	100	30	96.77	
Yes	0	0	1	3.23	
**Wound infection**					<0.001 ***
No	21	70.0	7	22.6	
Yes	9	30.0	24	77.4	
**Sepsis**					0.021 *
No	26	86.7	18	58.1	
Yes	4	13.3	13	41.9	
**Hospitalization days**	51.80 ± 31.77		76.81 ± 50.63		0.025 *

Data are expressed as the number (%) or mean ± standard deviation (SD), as appropriate. * *p* < 0.05, *** *p* < 0.001, by Chi-square test, Fisher’s exact test, or Student’s *t*-test, as appropriate.

**Table 2:** The risks for wound infection and sepsis in all patients.

	Wound Infection			Sepsis		
Statistics	*p*-Value	OR	95% CI	*p*-Value	OR	95% CI
**Age (years)**	0.180	0.90	0.76–1.05	0.365	0.89	0.70–1.14
**BMI (kg/m^2^)**	0.863	1.04	0.69–1.55	0.791	1.09	0.58–2.05
**TBSA of burns (%)**	0.540	1.01	0.98–1.05	0.006 **	1.09	1.03–1.16
**Sex gender**						
Male	reference			reference		
Female	0.313	0.509	0.14–1.90	0.911	1.10	0.21–5.89
**Groups**						
Control group	reference			reference		
Supplement group	0.002 **	0.11	0.03–0.43	0.014 *	0.09	0.01–0.61

* *p* < 0.05, ** *p* < 0.01, logistic regression analysis of adjusted odds ratio (OR) and 95% confidence interval (CI) for the occurrence of wound infection and sepsis, compared with the reference group.
